# Supplementary material for: End Sequence Analysis Toolkit (ESAT) expands the extractable information from single-cell RNA-seq data
Source: Genome Res. 2016 Oct;26(10):1397–410. doi: 10.1101/gr.207902.116 (PMC5052061; doi:10.1101/gr.207902.116)
Supplement: Supplemental Material [file supp_gr.207902.116_Supp_Materials.tar.gz › ESAT-esat_v0.1/doc/html/functions_func_i.html]

ESAT: Class Members - Functions


ESAT


- Main Page
- Related Pages
- Packages
- Classes
- Files

- Class List
- Class Index
- Class Hierarchy
- Class Members

- All
- Functions
- Variables
- Enumerator

- a
- b
- c
- d
- e
- f
- g
- h
- i
- j
- k
- l
- m
- n
- o
- p
- q
- r
- s
- t
- u
- v
- w
- z


All Classes Namespaces Files Functions Variables Enumerator Pages

### - i -

- ic()
  : broad.core.motif.PositionWeightMatrix
- identity()
  : Jama.Matrix
- IllegalTreeOperationException()
  : broad.core.datastructures.IllegalTreeOperationException
- IncompleteMethodImplementationException()
  : umms.core.exception.IncompleteMethodImplementationException
- IncrementScoreIfOverlap()
  : broad.core.annotation.AnnotationReader< T extends GenomicAnnotation >
  , broad.pda.annotation.BEDFileParser
- init()
  : broad.core.annotation.AnnotationReader< T extends GenomicAnnotation >
  , broad.core.motif.PositionWeightColumn
  , broad.pda.seq.segmentation.AlignmentDataModelStats
- initColIndexMaps()
  : broad.core.datastructures.MatrixWithHeaders
- initFromGene()
  : umms.core.annotation.Gene
- initFromIGVMatrix()
  : broad.pda.datastructures.LocationAwareMatrix
- initFromNanostring()
  : broad.core.datastructures.MatrixWithHeaders
- initFromReader()
  : broad.core.datastructures.MatrixWithHeaders
- initFromRegularMatrix()
  : broad.core.datastructures.MatrixWithHeaders
- initGCTFromReader()
  : broad.core.datastructures.MatrixWithHeaders
- initializeFilterRejectWriters()
  : broad.pda.seq.protection.MultiSampleScanPeakCaller
- initNameIndexMaps()
  : broad.core.datastructures.MatrixWithHeaders
- initRegion()
  : umms.core.model.score.WindowProcessor< S extends WindowScore >.AbstractProcessor< S extends WindowScore >
  , umms.core.model.score.WindowProcessor< S extends WindowScore >
- initRowDescrIndexMaps()
  : broad.core.datastructures.MatrixWithHeaders
- initRowIndexMaps()
  : broad.core.datastructures.MatrixWithHeaders
- inOrientedDegree()
  : broad.pda.seq.graph.ChromosomeWithBubblesJGraphT
- inReversedOrientation()
  : broad.core.annotation.BasicLightweightAnnotation
  , broad.core.annotation.LightweightGenomicAnnotation
  , broad.core.sequence.SequenceRegion
- insert()
  : broad.pda.feature.genome.Chromosome
  , broad.pda.feature.genome.DirectoryInstalledGenomeAssembly
- intersect()
  : broad.core.annotation.AnnotationReader< T extends GenomicAnnotation >
  , broad.core.annotation.BasicLightweightAnnotation
  , broad.core.sequence.SequenceRegion
  , broad.pda.annotation.BEDFileParser
  , umms.core.annotation.AbstractAnnotation
  , umms.core.annotation.Annotation
  , umms.core.annotation.AnnotationList< T extends Annotation >
  , umms.core.annotation.BasicAnnotation
  , umms.core.annotation.CompoundInterval
  , umms.core.annotation.SingleInterval
- IntersectBlocks()
  : broad.core.annotation.BED
- intersectGeneSets()
  : broad.pda.gene.GeneTools
- introduceGaps()
  : broad.core.multiplealignment.MultipleAlignment
- inverse()
  : Jama.Matrix
- invert()
  : broad.pda.feature.genome.Chromosome
  , broad.pda.feature.genome.DirectoryInstalledGenomeAssembly
- isAddToReadName()
  : broad.pda.seq.fastq.FastqSplitterByBarcode
- isAdjacent()
  : broad.core.datastructures.Interval
- isAlignmentPaired()
  : broad.pda.seq.alignment.StoredAlignmentStats
- isAtEnd()
  : broad.pda.seq.fastq.FastqSplitterByBarcode
- isAutosome()
  : broad.pda.feature.genome.Chromosome
- isChimera()
  : umms.core.alignment.AbstractPairedEndAlignment
  , umms.core.alignment.Alignment
  , umms.core.alignment.SingleEndAlignment
- isCodingGene()
  : umms.core.annotation.Gene
- isColumnNonTrivial()
  : broad.core.datastructures.MatrixWithHeaders
- isComplete()
  : umms.core.alignment.AlignmentPair
- isControl()
  : broad.pda.geneexpression.ExpressionExperimentInfo
- isControlProbe()
  : broad.pda.geneexpression.agilent.AgilentProbe
- isDiagonal()
  : Jama.Matrix
- isDirect()
  : broad.core.annotation.BasicTwoSubjectAnnotation
  , broad.core.annotation.TwoSubjectAnnotation
- isDuplicate()
  : umms.core.alignment.AbstractPairedEndAlignment
  , umms.core.alignment.Alignment
  , umms.core.alignment.SingleEndAlignment
- isEmpty()
  : broad.core.datastructures.IntervalTree< V >
  , broad.core.datastructures.Pair< T1 >
  , broad.core.motif.PositionWeightMatrix
  , broad.core.multiplealignment.MAFAlignment
  , broad.core.multiplealignment.MultipleAlignment
  , broad.pda.annotation.BEDFileParser
- isEnough()
  : broad.core.annotation.GenomicAnnotationFilter< T extends LightweightGenomicAnnotation >
- IsExactOverlappingIsoform()
  : broad.pda.gene.GeneWithIsoforms
- isExpressed()
  : broad.pda.seq.clip.GenomeSpaceSampleData
  , broad.pda.seq.clip.SampleData
  , broad.pda.seq.protection.GenomeSpaceSampleData
  , broad.pda.seq.protection.MultiSampleScanPeakCaller
  , broad.pda.seq.protection.SampleData
- isExpressedInAllControlSamples()
  : broad.pda.seq.protection.MultiSampleScanPeakCaller
- isFlagged()
  : broad.pda.geneexpression.agilent.AgilentProbe
- isFlankedBy()
  : broad.core.annotation.BasicGenomicAnnotation
  , broad.core.annotation.GenomicAnnotation
  , broad.core.sequence.SequenceRegion
- isForwardStrand()
  : broad.core.sequence.Sequence
- isFullRank()
  : Jama.QRDecomposition
- isFullyComaptible()
  : broad.pda.annotation.Locus
- isFullyCompatible()
  : umms.core.annotation.Gene
- isGap()
  : broad.core.sequence.Sequence
  , broad.pda.seq.graph.ChromosomeWithBubblesJGraphT
- isGenomicSpace()
  : broad.pda.seq.rap.GenomeCommandLineProgram
- isInAllGaps()
  : broad.pda.seq.graph.ChromosomeWithBubblesJGraphT
- isInExon()
  : umms.core.annotation.Gene
- isJnlp()
  : jaligner.util.Commons
- isMapped()
  : umms.core.alignment.AbstractPairedEndAlignment
  , umms.core.alignment.Alignment
  , umms.core.alignment.SingleEndAlignment
- isNegativeStrand()
  : broad.core.sequence.SequenceRegion
  , umms.core.alignment.AbstractPairedEndAlignment
  , umms.core.alignment.Alignment
  , umms.core.annotation.AbstractAnnotation
  , umms.core.annotation.Annotation
  , umms.core.annotation.Gene
- isNegativeStranded()
  : broad.pda.seq.segmentation.AlignmentDataModel
  , broad.pda.seq.segmentation.GenericAlignmentDataModel
- isNonsingular()
  : Jama.LUDecomposition
- isoformMapContains()
  : umms.core.scripture.BuildScriptureCoordinateSpace
- isOverlapCompatible()
  : broad.pda.annotation.BEDFileParser
- isPaired()
  : broad.pda.seq.fastq.FastqSplitterByBarcode
  , broad.pda.seq.segmentation.AlignmentDataModelStats
  , umms.core.alignment.AbstractPairedEndAlignment
  , umms.core.alignment.Alignment
  , umms.core.alignment.SingleEndAlignment
- isPairedEndFormat()
  : umms.core.readers.PairedEndReader
- isPartialPolyA()
  : broad.pda.seq.fastq.FastqSequence
- isPCRLike()
  : broad.core.primer3.isPCRLike
- isPolyA()
  : broad.pda.seq.fastq.FastqSequence
- isPositiveStranded()
  : broad.pda.seq.segmentation.AlignmentDataModel
  , broad.pda.seq.segmentation.GenericAlignmentDataModel
- isProbeFound()
  : broad.pda.geneexpression.agilent.AgilentProbe
- isProbeNonUniform()
  : broad.pda.geneexpression.agilent.AgilentProbe
- isProbePopulationOutlier()
  : broad.pda.geneexpression.agilent.AgilentProbe
- isProbeWellAboveBackground()
  : broad.pda.geneexpression.agilent.AgilentProbe
- isProperPair()
  : umms.core.alignment.AbstractPairedEndAlignment
  , umms.core.alignment.Alignment
  , umms.core.alignment.SingleEndAlignment
- isRefGapped()
  : broad.core.multiplealignment.MultipleAlignment
- isSafeToLoad()
  : broad.pda.seq.alignment.StoredAlignmentStats
- isSaturated()
  : broad.pda.geneexpression.agilent.AgilentProbe
- isSexChromosome()
  : broad.pda.feature.genome.Chromosome
- isSpanningGap()
  : broad.pda.seq.graph.ChromosomeWithBubblesJGraphT
- isSPD()
  : Jama.CholeskyDecomposition
- isStopCodon()
  : umms.core.annotation.Gene
- isStranded()
  : broad.pda.seq.segmentation.AlignmentDataModel
  , broad.pda.seq.segmentation.GenericAlignmentDataModel
- isSymmetric()
  : Jama.Matrix
- isTerminiCoverageBias()
  : broad.pda.annotation.Locus
- isToComplex()
  : broad.pda.seq.graph.Path
- isUnoriented()
  : broad.core.sequence.SequenceRegion
  , umms.core.annotation.Annotation
  , umms.core.annotation.BasicAnnotation
- isValid()
  : umms.core.model.AlignmentModel
  , umms.core.model.JCSAlignmentModel
- isValidWindow()
  : umms.core.coordinatesystem.CoordinateSpace
  , umms.core.coordinatesystem.GenomicSpace
  , umms.core.coordinatesystem.MaskedGenomicSpace
  , umms.core.coordinatesystem.TranscriptInGenomicSpace
  , umms.core.coordinatesystem.TranscriptomeSpace
- iterator()
  : broad.core.datastructures.IntervalTree< V >
  , broad.pda.samtools.SAMPairedEndFileReader
  , broad.pda.seq.graph.ChromosomeWithBubblesJGraphT
  , umms.core.annotation.AnnotationCollection< T extends Annotation >
  , umms.core.annotation.AnnotationList< T extends Annotation >
  , umms.core.model.AlignmentModel
  , umms.core.model.JCSAlignmentModel
  , umms.core.readers.PairedEndReader


---

Generated on Fri Aug 15 2014 15:11:16 for ESAT by  

 1.8.7
